# Integrated mineralogical, geochemical, and log derived TOC evaluation of source rock potential in the Khatatba Formation (Western Desert, Egypt)

**DOI:** 10.1038/s41598-026-57273-z

**Published:** 2026-06-18

**Authors:** Shahenda M. Khalaf, Mohamed M. Abdel Fattah, Mohamed I. Abdel-Fattah, Hamdan A. Hamdan, Zakaria M. Abd-Allah

**Affiliations:** 1 Geology Department, Faculty of Science, Beni-Suef University, Beni Suef, Egypt; 2 Petroleum Geosciences & Remote Sensing Program, Department of Applied Physics and Astronomy, College of Sciences, University of Sharjah, Sharjah, UAE; 3 Geology Department, Faculty of Sciences, Suez Canal University, Ismailia, Egypt

**Keywords:** Source rock evaluation, Log-derived TOC, Rock-eval pyrolysis, Mineralogy and geochemistry, Obaiyed field, Khatatba formation, Environmental sciences, Solid Earth sciences

## Abstract

The Middle Jurassic Khatatba Formation represents one of the principal petroleum system elements in the Western Desert of Egypt and contains important organic-rich shale intervals within the Upper Safa Member. This study integrates mineralogical, geochemical, and calibrated log-derived TOC analyses to evaluate the source rock potential of the Upper Safa Member in the Obaiyed Field. A total of 100 ditch-cutting samples from four wells were examined using XRD, XRF, TOC, TS, and Rock-Eval pyrolysis analyses, combined with continuous log-derived TOC prediction. Mineralogical results indicate a kaolinite-rich siliciclastic assemblage with quartz and minor calcite, while geochemical data suggest mixed siliciclastic and marine influence. TOC values range from 0.50 to 3.90 wt% (average ~ 1.8 wt%), indicating moderate to very good organic richness. Rock-Eval data reveal mixed Type II/III kerogen with HI values of 99–186 mg HC/g TOC and Tmax values between 441 °C and 457 °C, confirming thermal maturity within the hydrocarbon generation window. Log-derived TOC calibrated against laboratory measurements shows strong agreement (R² = 0.93), demonstrating the reliability of the integrated workflow for continuous source rock characterization. TOC–TS relationships suggest deposition under oxygen-restricted conditions interpreted conservatively as dysoxic to suboxic, favorable for organic matter preservation. The Upper Safa Member is interpreted as a mature, gas-prone source rock with fair to good hydrocarbon generation potential and strong source–reservoir coupling with adjacent Safa sandstone reservoirs. The integrated workflow presented in this study provides a reliable framework for continuous source rock evaluation and improved exploration targeting in the northwestern Western Desert of Egypt.

## Introduction

**Fig. 1:**
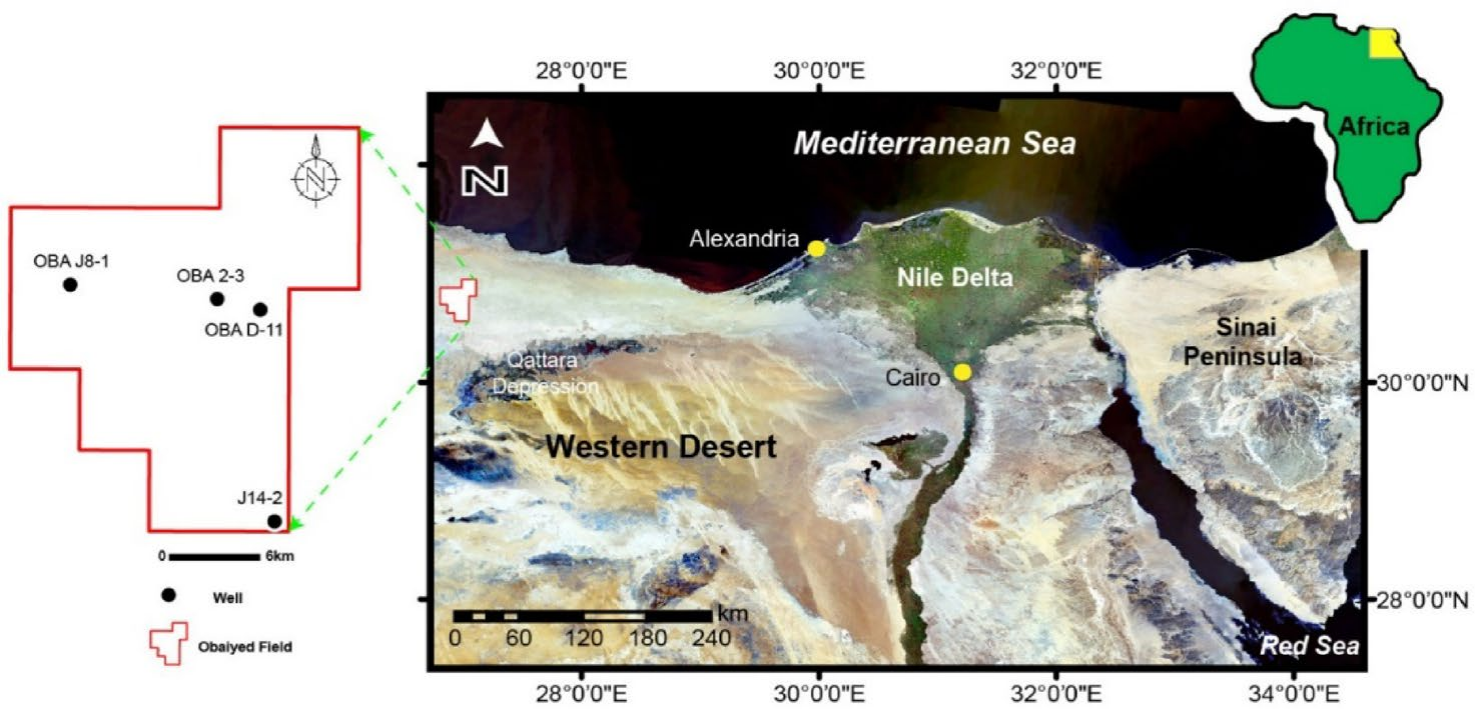
Location of the Obaiyed Field and the studied wells in the northwestern Western Desert, Egypt. Map was generated using ArcGIS software version 2025 (https://www.esri.com/en-us/arcgis/geospatial-platform/overview).

The Western Desert of Egypt is one of the most prolific hydrocarbon-producing regions in North Africa, supplying over half of the country’s oil and gas reserves and continuing to attract strong exploration interest^1–6^. The most promising Jurassic petroleum systems within this basin are associated with the Middle Jurassic clastic successions, particularly the Khatatba Formation, which plays a dual role as both a source rock and a reservoir^7–9^. The Obaiyed Field (Fig. 1), located in the northwestern part of the Western Desert near the Qattara Depression, represents one of the major gas-producing areas in this sector, where production is primarily derived from the Upper and Lower Safa Members of Khatatba Formation —high-quality sandstone intervals interbedded with organic-rich marine shales^10–13^. This close vertical relationship supports the development of a self-sourcing petroleum system characterized by short-distance hydrocarbon migration and strong source–reservoir coupling.

Effective evaluation of the Khatatba shale intervals is therefore essential for reconstructing the generative history and predicting hydrocarbon charge scenarios. Source rocks are typically assessed on the basis of their mineralogical composition, organic matter abundance, kerogen type, and maturity level—parameters that directly influence generation potential and expelled hydrocarbon phase^14–16^. Mineralogy helps infer provenance and depositional environment, while major and trace elements serve as proxies for redox conditions that control organic matter preservation^17–20^. Quantitative geochemical indicators such as Total Organic Carbon (TOC), Total Sulfur (TS), and Rock-Eval pyrolysis indices provide detailed insights into source rock quality, expected hydrocarbon type, and thermal evolution^21–23^.

Despite the recognized significance of the Khatatba Formation in regional petroleum systems, previous studies have largely focused on broader basin-scale interpretations, with limited attention to localized source intervals in the Obaiyed Field^7,8,16^. Moreover, traditional source rock assessments rely heavily on core and cutting sampling, which are spatially discontinuous and often inadequate for capturing vertical heterogeneity across thick stratigraphic successions. Recent advancements in petrophysical modeling have introduced log-derived TOC estimation methods—such as the ΔLogR technique and multi-parameter TOC transforms—that provide continuous high-resolution profiling of organic richness^24–28^. When calibrated with laboratory data, these approaches significantly improve source rock prediction in both data-rich and data-limited settings.

The novelty of this study lies in integrating mineralogical, geochemical, and log-derived TOC analyses within a single calibrated workflow for source rock evaluation of the Khatatba Formation. Unlike previous studies that relied mainly on conventional laboratory analyses or regional interpretations, this work combines laboratory measurements with statistically validated petrophysical TOC models to achieve continuous characterization of organic richness. The study also addresses the limitation of discontinuous core and cutting data in capturing vertical heterogeneity within the Upper Safa Member. In addition, multiple TOC prediction methods were comparatively evaluated to improve the reliability of log-derived TOC estimation. Therefore, this study represents more than a conventional local source rock assessment by providing an integrated and reproducible workflow for continuous source rock characterization and improved understanding of source–reservoir relationships in the Khatatba petroleum system. Furthermore, the integrated datasets and interpretations presented in this study provide an important baseline reference for future investigations of the Khatatba Formation as an unconventional shale gas reservoir, particularly for studies related to rock mechanics, geomechanics, and hydraulic fracturing applications.

## Geological background

The Western Desert of Egypt comprises a series of intracratonic basins developed along the northern African passive margin, forming one of the country’s most productive hydrocarbon provinces^1,3,4,6^. The study area, the Obaiyed Field, lies within the northwestern Desert close to the Qattara Depression, where Jurassic stratigraphy records extensive syn-rift and post-rift sag-phase sedimentation controlled by the opening of the Neotethys Ocean^10,21,29^.

**Fig. 2:**
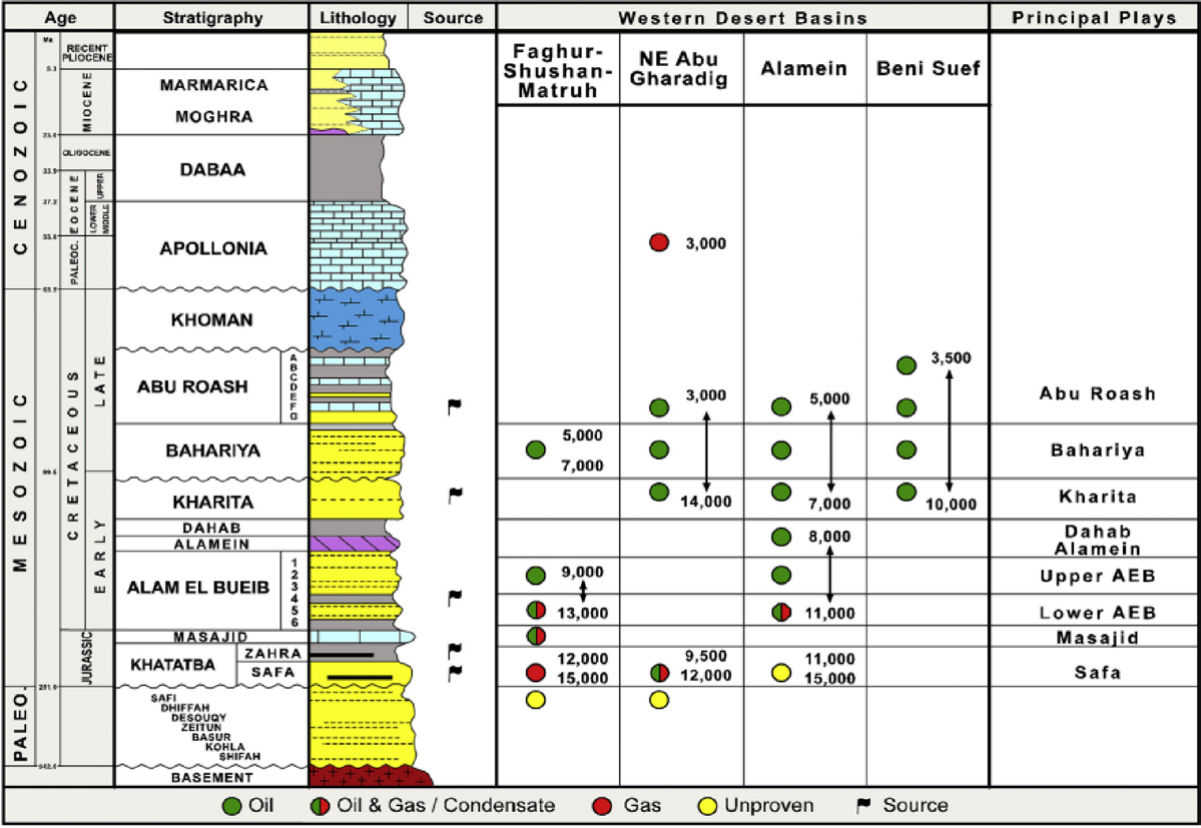
Simplified stratigraphic framework of the Western Desert highlighting major lithologic units, identified source rock intervals, and principal hydrocarbon plays across key sub-basins^9^.

Within this regional framework, the Middle Jurassic Khatatba Formation represents a major petroleum system element across the Matruh and Shushan sub-basins (Fig. 2). The formation consists of alternating sandstone, siltstone, and shale units, reflecting deposition in shallow marine to deltaic environments with strong siliciclastic influx^18,30^. The Khatatba Formation consists of four members: Zahra, Upper Safa, Kabrit, and Lower Safa. The Upper and Lower Safa Members contain extensive fine-grained intervals rich in organic matter that have acted as key source rocks for gas accumulations in nearby reservoirs^18,31^. Interbedded fluvial-deltaic sandstones form excellent reservoir horizons, supported by intra-formational seals that reduce migration distances and promote efficient hydrocarbon retention^9,16,29^. The Zahra Member is mainly composed of shale with minor silty and calcareous interbeds and serves as an effective regional top seal, whereas the Kabrit Member consists predominantly of evaporitic and carbonate-rich lithologies with interbedded shale and siltstone, forming a low-permeability seal that separates the Lower and Upper Safa reservoir units within the Khatatba Formation of the northwestern Western Desert, Egypt^16^.

Rifting in the Middle Jurassic produced numerous fault-bounded depocenters, which provided accommodation space for thick sediment accumulation and enhanced burial of organic-rich shales. Subsequent Cretaceous subsidence and Late Cretaceous–Cenozoic compressional tectonism contributed to thermal maturation of the Khatatba source intervals into the main gas generation window^21,32,33^. Structural traps are typically associated with normal fault blocks and rollover anticlines aligned with regional extensional structural trends.

## Materials and methods

### Materials

A total of one hundred (100) ditch-cutting samples were collected from four wells (OBA 2–3, J14-2, OBA J8-1, and OBA D11) penetrating the Khatatba Formation in the Obaiyed Field, Western Desert, Egypt (Fig. 1), with sampling facilitated by Badr El-Din Petroleum Company (Bapetco). These samples cover the full stratigraphic succession of the formation, including the Zahra, Upper Safa, Kabrit, and Lower Safa members, and represent a wide range of depths and lithological characteristics. Given that the focus of this study is source rock evaluation, the analysis was restricted to shale-dominated intervals within the Upper Safa Member, identified using lithological descriptions and gamma-ray log responses. Accordingly, twenty (20) representative shale samples were selected for TOC analysis to capture vertical variability in organic richness, from which twelve (12) samples with TOC values greater than 0.5 wt% were further analyzed for Rock-Eval pyrolysis and total sulfur, following standard geochemical practice. In addition, ten (10) samples were selected for detailed mineralogical and geochemical characterization using XRD and XRF techniques. To minimize uncertainties inherent to ditch-cutting samples, including cavings, depth mismatch, and mixing, quality control procedures were implemented, including log-based depth matching, consistency checks with lithological descriptions, and exclusion of intervals affected by borehole instability.

In addition to physical core and cutting materials, a comprehensive suite of wireline logs was acquired from the same wells to complement laboratory measurements and provide continuous depth-based assessment of organic richness. The log dataset includes gamma ray (GR), deep and shallow resistivity (RES_deep, RES_shallow), bulk density (RHOB), neutron porosity (NPHI), sonic transit time (DT), and photoelectric factor (PEF). These logs were essential for deriving multiple TOC estimation curves—such as TOC_DEN, TOC_DT, TOC_RHOB, TOC_NPHI, and ΔLogR-dervied TOC—which were subsequently calibrated against laboratory-measured TOC values to ensure accuracy and minimize interpretation uncertainty.

### Methods

#### Laboratory analyses

Mineralogical and geochemical analyses were performed to evaluate the mineral composition and depositional characteristics of the Khatatba Formation (Upper Safa Member) shales. XRD was employed to determine the crystalline mineral phases, with powdered samples analyzed using a PANalytical Empyrean (model 202960) diffractometer equipped with a Cu-Kα radiation source (λ = 1.5406 Å), operating at 40 kV and 30 mA. The scans covered a 2θ range from 5° to 80°, with a step size of 0.02° and 0.5-second counting time per step, enabling precise identification of both clay and non-clay components. Mineral phases were determined by matching the obtained diffraction peaks with standard reference patterns^34^, ensuring reliable mineralogical interpretation. Complementary geochemical analysis was conducted using XRF to quantify major and trace oxide concentrations that reflect sediment provenance and depositional influences^35–38^. For this purpose, finely powdered samples were homogenized with a binder and pressed into pellets prior to measurement using a Philips PW 1404 wavelength-dispersive spectrometer, which provided accurate quantification of key oxides such as SiO₂, Al₂O₃, CaO, Fe₂O₃, MgO, Na₂O, and selected trace constituents^14^. The combined application of XRD and XRF offers an integrated assessment of the shale mineral assemblage and chemical composition, allowing interpretation of siliciclastic input, carbonate contribution, and early diagenetic processes^39,40^. These analyses provide critical baseline information for understanding the depositional environment and evaluating the potential influence of mineralogy and geochemistry on organic matter preservation and source rock quality within the Khatatba Formation^18,21,41^.

Organic geochemical parameters were obtained through TOC, TS, and Rock-Eval pyrolysis analyses. TOC measurements were performed using a LECO C230 analyzer after removing inorganic carbonates with hydrochloric acid^42^. TS values were determined using a LECO SC632 sulfur analyzer, in which sulfur content was quantified from SO₂ produced during combustion^43,44^. Rock-Eval pyrolysis^17^ was conducted on approximately 0.15 g of powdered sample to determine S1, S2, S3, T_max_, HI, OI, and PI, enabling assessment of kerogen type, organic matter quality, and thermal maturity^6,45,46^.

#### TOC prediction methods

Log-derived TOC was calculated using several established petrophysical methods to provide continuous TOC profiles across the Khatatba Formation. These include: (i) the ΔLogR method, which uses the separation between resistivity and sonic logs to quantify TOC in thermally mature intervals^24,25,28^; (ii) density-derived TOC (TOC_DEN) and multi-parameter transforms integrating RHOB, NPHI, and DT^47^; and (iii) resistivity-integrated TOC algorithms calibrated for organic-rich shales^1,48^. Each TOC curve was calibrated against laboratory-measured TOC values to ensure consistency, reduce uncertainty, and provide reliable high-resolution estimation of organic richness across the Upper Safa Member.

log-derived TOC was quantified using both physics-based and empirical approaches, followed by systematic calibration against laboratory-measured TOC values. The ΔLogR technique of Passey et al. (1990) was applied as the primary method for TOC estimation in thermally mature intervals. The separation between resistivity and sonic logs was calculated as:

$$\:{\Delta\:}\mathrm{l}\mathrm{o}\mathrm{g}R={\mathrm{l}\mathrm{o}\mathrm{g}}_{10}\left(\frac{R}{{R}_{baseline}}\right)+0.02\left({\Delta\:}t-{\Delta\:}{t}_{baseline}\right)$$

where $\:R$is deep resistivity (ohm·m), $\:{\Delta\:}t$is sonic transit time (µs/ft), and baseline values represent non-source shale intervals. Baseline trends were defined for each well using low-GR, low-TOC intervals to minimize lithological bias.

TOC was then computed using:

$$\:TOC={\Delta\:}\mathrm{l}\mathrm{o}\mathrm{g}R\times\:{10}^{\left(2.297-0.1688\times\:LOM\right)}$$

where LOM (Level of Organic Maturity) was estimated from Rock-Eval Tmax values using established correlations. For the studied intervals (Tmax = 441–457 °C), LOM values between 8 and 10 were adopted, consistent with early–peak gas window maturity.

A density-based approach was implemented to estimate TOC using the relationship:

$$\:TOC=\frac{{\rho\:}_{matrix}-{\rho\:}_{bulk}}{{\rho\:}_{matrix}-{\rho\:}_{organic}}\times\:100$$

where $\:{\rho\:}_{bulk}$is measured bulk density (RHOB), $\:{\rho\:}_{matrix}$is matrix density (assumed 2.65 g/cm³ for siliciclastic rocks), and $\:{\rho\:}_{organic}$is organic matter density (1.2–1.4 g/cm³). A value of 1.3 g/cm³ was adopted based on literature for Type II/III kerogen.

Additional TOC estimates were generated using multi-log regression models incorporating RHOB, NPHI, and DT logs. These models were developed through linear and non-linear regression against measured TOC:

$$\:TOC=a+b\left({\rho\:}_{b}\right)+c\left({\phi\:}_{N}\right)+d\left({\Delta\:}t\right)$$

where $\:a,b,c,$and $\:d$are regression coefficients derived from calibration datasets. Separate models were tested for each well to account for local lithological variations.

#### Calibration strategy

All log-derived TOC curves were calibrated against laboratory TOC measurements obtained from 100 cutting samples (with representative subsets analyzed geochemically). Depth matching between log data and cutting samples was carefully corrected to account for lag effects and mixing inherent to ditch cuttings. Calibration was performed using least-squares regression, minimizing residual error between measured and log-derived TOC. Outliers associated with poor hole conditions or suspected cavings were excluded based on log quality control (e.g., caliper enlargement, inconsistent density–neutron response).

All log-derived TOC curves were calibrated against laboratory TOC measurements obtained from 20 cutting samples (with representative subsets analyzed geochemically for Upper Safa Member). Depth matching between log data and cutting samples was carefully corrected to account for lag effects and mixing inherent to ditch cuttings. Calibration was performed using least-squares regression, minimizing residual error between measured and predicted TOC. Outliers associated with poor hole conditions or suspected cavings were excluded based on log quality control (e.g., caliper enlargement, inconsistent density–neutron response).

#### Statistical evaluation

To quantitatively evaluate the performance and robustness of the log-derived TOC prediction methods, a comparative statistical analysis was conducted using laboratory-measured TOC data from 20 samples collected from four wells within the Upper Safa Member. The dataset was randomly divided into training (70%) and testing (30%) subsets to allow independent model calibration and validation, while ensuring representation of lithological variability across the study area. Log-derived TOC was estimated using the ΔLogR method together with additional petrophysical-based approaches, including density-derived TOC (TOC_DEN), neutron-derived TOC (TOC_NPHI), and sonic-derived TOC (TOC_DT). Prior to calibration, depth matching corrections between cutting samples and wireline logs were performed, and intervals affected by poor hole conditions or suspected cavings were excluded through quality-control procedures. Cross-validation was also applied during model calibration to reduce overfitting and assess model stability. The predictive performance and uncertainty of each method were evaluated using the coefficient of determination (R²), root mean square error (RMSE), mean absolute error (MAE), and mean absolute percentage error (MAPE), calculated by comparing predicted TOC values with laboratory-measured TOC data. These procedures provide a more rigorous assessment of the reliability and uncertainty of the log-derived TOC prediction workflow.

## Results

### Mineralogical composition

**Fig. 3:**
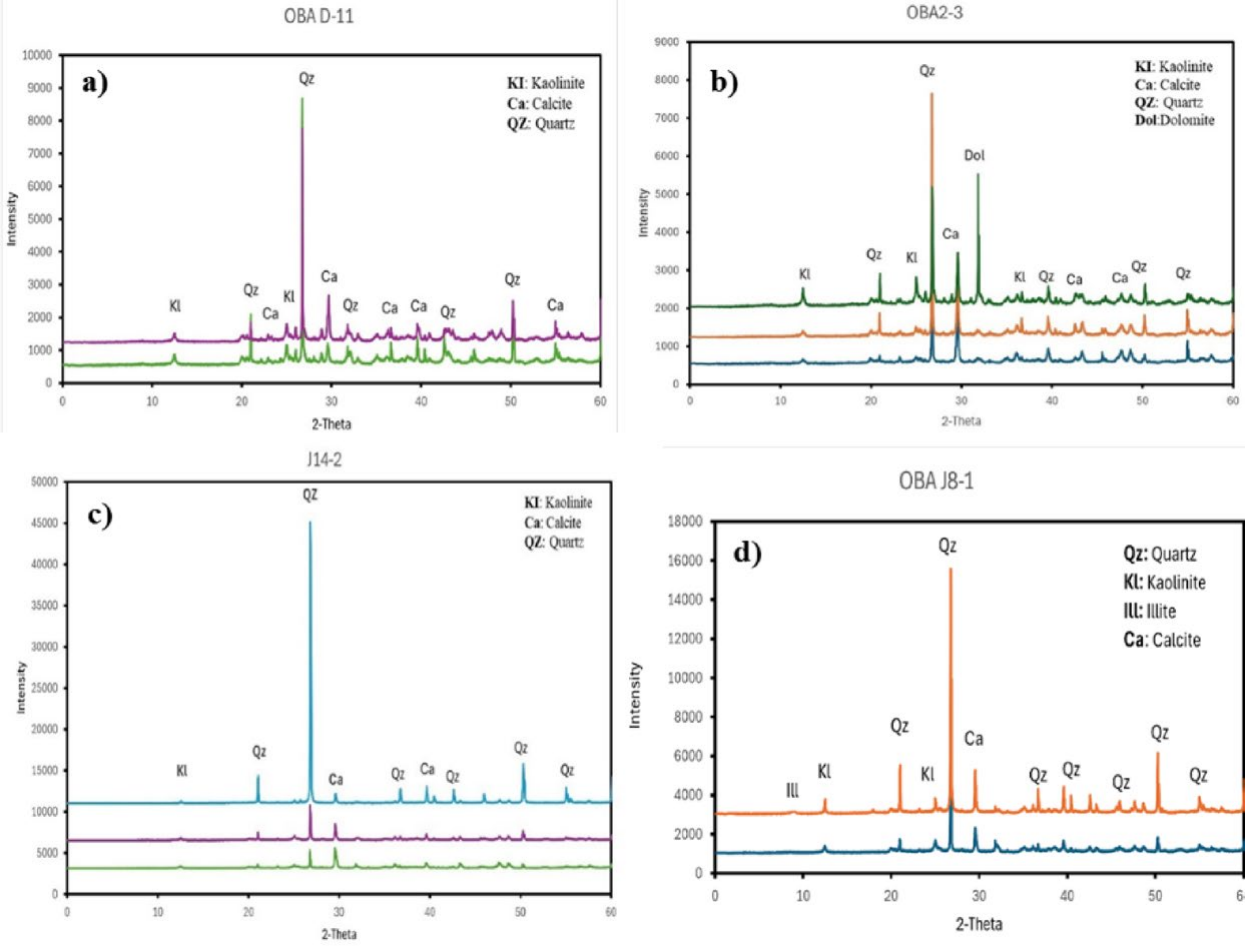
XRD patterns of Khatatba Formation shale samples from the Obaiyed Field wells: **(a)** OBA D-11, **(b)** OBA 2–3, **(c)** J14-2, and **(d)** OBA J8-1, showing dominant mineral phases including kaolinite (Kl), quartz (Qz), calcite (Ca), dolomite (Dol), and illite (Ill).

XRD analysis of the Khatatba Formation (Upper Safa Member) shales reveals a mineral composition dominated by kaolinite, with quartz and calcite as the dominant non-clay minerals (Table 1; Fig. 3). The high kaolinite content suggests intensive chemical weathering of continental source rocks and significant terrigenous influx into the basin, typical of humid climatic settings and shallow-marine depositional environments^10,41^. Quartz abundance further supports a siliciclastic detrital origin, while the associated calcite may reflect periodic marine conditions contributing minor carbonate material^18^. The absence of chlorite in all examined samples indicates limited burial diagenesis and low-temperature alteration, favoring kaolinitization over chloritization^21^. Overall, the mineralogical characteristics are consistent with deposition under mixed marine–continental conditions and align well with previous interpretations of the Upper Safa Member as a shallow-marine siliciclastic system influenced by strong terrestrial input^14,15,49^.

**Table 1:** Major and trace oxide compositions (wt%) of Khatatba Formation (Upper Safa Member) shale samples from the Khatatba Formation in the Obaiyed Field (Western Desert, Egypt).

Compound Formula	Concentration (wt%)										
	(1) 2056/24 (A1)	(2) 2057/24 (A3)	(3) 2058/24 (A5)	(4) 2059/24 (B1)	(5) 2060/24 (B3)	(6) 2061/24 (B5)	(7) 2062/24 (C3)	(8) 2063/24 (C4)	(9) 2064/24 (D3)	(10) 2065/24 (D5)	Average
**Na**_**2**_ **O**	0.618	0.782	0.272	1.674	1.317	1.032	1.772	1.196	0.735	0.402	0.98
**MgO**	0.618	0.532	0.189	0.821	0.750	0.639	0.684	0.880	0.582	0.462	0.61
**Al**_**2**_ **O**_**3**_	9.866	11.369	2.665	10.289	9.443	11.049	15.513	15.257	15.267	10.993	11.17
**SiO**_**2**_	20.976	29.525	63.361	20.251	20.690	23.362	39.041	31.209	33.889	39.049	32.13
**P**_**2**_ **O**_**5**_	0.116	0.102	0.043	0.112	0.106	0.085	0.106	0.114	0.116	0.112	0.1
**SO**_**3**_	1.624	1.367	1.640	1.416	1.882	1.791	1.933	2.418	1.234	2.203	1.75
**K**_**2**_ **O**	0.974	0.849	0.204	1.081	0.845	1.147	1.026	1.085	1.470	2.310	1.09
**CaO**	22.142	14.784	7.112	19.317	15.870	12.276	7.016	11.319	9.658	11.276	13.07
**TiO**_**2**_	0.990	1.119	0.319	0.778	0.898	1.075	0.914	0.768	1.288	1.138	0.92
**Cr**_**2**_ **O**_**3**_	-----	-----	-----	0.025	0.027	0.025	-----	-----	-----	0.025	0.02
**MnO**	0.194	0.201	0.047	0.193	0.127	0.122	0.122	0.107	0.176	0.077	0.13
**Fe**_**2**_ **O**_**3**_^**tot**^	12.083	9.342	2.033	9.504	9.545	9.495	6.194	7.032	10.044	4.621	7.98
**Co**_**3**_ **O**_**4**_	-----	0.021	-----	0.018	0.020	0.022	-----	-----	0.021		0.02
**ZnO**	0.012	0.015	-----	0.010	0.017	0.015	0.009	0.009	0.014	0.008	0.01
**Rb**_**2**_ **O**	0.007	0.005	-----	0.005	0.005	0.007	0.004	0.005	0.008	0.010	0.01
**SrO**	0.042	0.026	0.020	0.041	0.041	0.062	0.056	0.072	0.030	0.035	0.04
**ZrO**_**2**_	0.043	0.050	0.020	0.027	0.039	0.045	0.040	0.034	0.053	0.057	0.04
**Nb**_**2**_ **O**_**5**_	0.005	0.005	-----	-----	0.004	0.005	0.003	0.004	0.007	-----	0.001
**BaO**	-----	0.029	0.043	0.589	0.661	1.777	2.140	2.755	0.057	0.100	0.9
**CeO**_**2**_	-----	0.044	-----	-----	-----	-----	-----	-----	0.057	-----	0.05
**PbO**	-----	-----	-----	-----	-----	-----	0.007	0.006	-----	-----	0.01
**Cl**	1.889	2.032	1.232	4.048	5.611	4.767	2.517	1.626	0.993	2.121	2.68
**Br**	-----	-----	-----	-----	-----	-----	0.005	0.004	-----	-----	0.01
**L.O.I**	27.8	27.80	20.80	29.80	32.10	31.20	20.9	24.10	24.30	25	26.38
**CIA**	59.45	70.99	71.01	39.01	63.08	72.14	79.56	75.83	77.45	67.73	67.63
**CIW**	63.16	74.96	75.09	63.16	66.86	77.99	82.64	80.16	83.69	78.97	74.67

### Major and trace oxides

XRF results show that SiO₂ (average 32.13 wt%), Al₂O₃ (11.17 wt%), and CaO (13.07 wt%) are the dominant oxides in the shale samples of the Upper Safa Member (Table 1). Elevated Fe₂O₃ (7.98 wt%) and moderate MgO (0.61 wt%) contents also characterize the succession. These compositional patterns reflect a mixed siliciclastic and biogenic carbonate influence, consistent with deposition in a shallow-marine shelf environment^36^. Trace oxide concentrations reveal notably high Ba (up to 0.9 wt%) and Cl (up to 2.68 wt%), with SrO and ZrO₂ averaging 0.04 wt% (Table 1). Positive correlations between SiO₂ and Al₂O₃, MgO, and Na₂O indicate a terrigenous provenance^17,50^, while SrO and ZrO₂ covariations support a combined siliciclastic–marine depositional regime. These geochemical signatures collectively support a sedimentary setting dominated by mixed continental and marine contributions.

### Richness of organic carbon and sulfur

The Khatatba Formation (Upper Safa Member) displays notable variability in organic richness, with TOC values ranging from 0.50 to 3.90 wt% and an overall average of approximately 1.8 wt% (Table 2;). These values exceed the commonly accepted threshold for productive source rocks and indicate that several intervals within the formation fall into the “very good” quality category for hydrocarbon generation potential. The highest TOC concentration was observed in well OBA J8-1 at a depth of 3850 m, suggesting enhanced preservation or localized enrichment of organic matter in that zone. Total sulfur values range between 0.49 and 1.98 wt%,

**Table 2:** TOC and Rock-Eval pyrolysis results for Khatatba Formation (Upper Safa Member) shales, including TOC, (TS, hydrocarbon yields (S1, S2, S3), kerogen quality indicators (HI, OI), maturity parameter (T_max_), and generation potential (PI and PY).

Well	Sample	Depth (m)	TOC	TS	S1	S2	S3	T_max_	HI	OI	PI	PY (s1 + s2)
	Type		wt%	wt%	mg/g	mg/g	mg/g					
**OBA 2–3**	CUT	3800	0.70	-----	-------	-------	-------	-------	------	------	------	------
	CUT	3830	1.95	1.88	0.11	2.09	1.91	456	107	98	0.05	2.2
	CUT	3850	0.75	-----	-------	-------	-------	-------	------	------	-----	----
	CUT	3880	2.11	0.87	0.12	2.47	3.24	450	117	154	0.05	2.59
	CUT	3910	1.74	1.18	0.11	1.73	1.96	457	99	113	0.06	1.84
**J14-2**	CUT	3905	0.77	-----	-------	-------	-------	-------	------	------	------	-------
	CUT	3930	1.39	0.81	0.15	2.17	2.81	441	156	202	0.06	2.32
	CUT	3950	1.46	0.49	0.16	2.72	2.83	443	186	194	0.06	2.88
	CUT	3980	1.79	0.66	0.15	2.88	2.50	447	161	140	0.05	3.03
	CUT	4120	0.50	-----	-------	-------	-------	-------	------	------	------	-----
**OBA D11**	CUT	3790	0.58	-----	-------	-------	-------	-------	------	------	------	------
	CUT	3820	0.82	0.85	0.09	1.42	2.69	441	173	328	0.06	1.51
	CUT	3850	1.98	1.98	0.11	2.44	3.66	449	123	185	0.04	2.55
	CUT	3880	1.04	1.58	0.09	1.15	2.07	443	111	199	0.07	1.24
	CUT	4045	0.46	-----	-------	-------	-------	-------	------	------	------	
**OBA J8-1**	CUT	3790	2.09	-----	-------	-------	-------	-------	------	------	------	-----
	CUT	3810	2.16	1.27	0.10	2.45	1.72	450	113	80	0.04	2.55
	CUT	3830	1.72	0.81	0.15	1.90	1.87	447	110	109	0.07	2.05
	CUT	3850	3.90	0.66	0.12	4.91	1.66	457	126	43	0.02	5.03
	CUT	3925	3.39	-----	-------	-------	-------	-------	------	------	------	-------

### Rock-eval pyrolysis

Rock-Eval pyrolysis conducted on twelve (12) Khatatba Formation (Upper Safa Member) shales with TOC values exceeding 0.5 wt% provides key parameters for evaluating organic matter quality and maturity (Table 2). The measured S1 values fall between 0.09 and 0.16 mg HC/g rock, indicating low free hydrocarbon content, while S2 values range from 1.15 to 4.91 mg HC/g rock, suggesting fair to good residual hydrocarbon generation potential.

These results align with the log-derived TOC distribution and reinforce the interpretation that the Upper Safa Member of Khatatba Formation serves as the principal source interval for the Obaiyed petroleum system.

### Log-derived TOC distribution

The integration of wireline logs—including GR, resistivity, RHOB, NPHI, DT, and PEF—enabled continuous TOC estimation across the Khatatba, Upper Safa, Kabrit, and Lower Safa intervals using multi-parameter and ΔLogR techniques. Log-derived TOC curves (TOC_DEN, TOC_DLRM, TOC_DLRAVG, and related transforms) reveal vertically variable organic richness that correlates well with laboratory measurements. The highest log-derived TOC responses coincide with thick shale intervals within the Upper Safa Member, especially where elevated resistivity and increased sonic transit times indicate the presence of organically enriched, compacted shale facies.

## Discussion

### Mineralogical characteristics and depositional conditions

Mineralogical results from the Upper Safa Member of Khatatba Formation show that kaolinite is the principal clay mineral accompanied mainly by quartz and calcite as dominant non-clay constituents (Fig. 3).

The interpretation of depositional conditions based on clay mineralogy should be considered with caution. Although the dominance of kaolinite and the absence of chlorite are commonly associated with intense chemical weathering and significant terrigenous input under humid conditions, these mineralogical signatures are not uniquely diagnostic and may also result from diagenetic processes or source rock composition^41^. Therefore, while the observed mineral assemblage is consistent with a siliciclastic system influenced by continental input, it does not independently constrain paleoclimate, sediment transport mechanisms, or burial history. More robust evaluation of weathering intensity and provenance would require additional geochemical proxies such as the Chemical Index of Alteration (CIA), the chemical index of weathering (CIW), rare earth element (REE) distributions, and isotopic analyses, which are beyond the scope of the present dataset. Accordingly, the interpretations presented here are expressed as plausible scenarios consistent with the available mineralogical evidence rather than definitive conclusions.

The chemical index of alteration [CIA] & the chemical index of weathering (CIW) can be used to determine the intensity chemical weathering^51^, where^52–54^:

1 $${\rm{CIA }} = {\rm{ }}\left[ {{\rm{A}}{{\rm{l}}_{\rm{2}}}{{\rm{O}}_{\rm{3}}}/{\rm{ }}\left( {{\rm{A}}{{\rm{l}}_{\rm{2}}}{{\rm{O}}_{\rm{3}}}\, + \,{\rm{CaO}}^*{\rm{ }} + {\rm{ N}}{{\rm{a}}_{\rm{2}}}{\rm{O}}\, + \,{{\rm{K}}_{\rm{2}}}{\rm{O}}} \right)} \right]{\rm{ x1}}00$$

2 $${\rm{CaO}}^* = {\rm{ mole CaO }} \times {\rm{ mole }}{{\rm{P}}_{\rm{2}}}{{\rm{O}}_{\rm{5}}} \times {\rm{ 1}}0/{\rm{5}}$$

3 $${\rm{CIW }} = {\rm{ }}\left[ {{\rm{A}}{{\rm{l}}_{\rm{2}}}{{\rm{O}}_{\rm{3}}}/{\rm{ }}\left( {{\rm{A}}{{\rm{l}}_{\rm{2}}}{{\rm{O}}_{\rm{3}}}\, + \,{\rm{CaO}}^*{\rm{ }} + {\rm{ N}}{{\rm{a}}_{\rm{2}}}{\rm{O}}} \right)} \right]{\rm{ }} \times {\rm{ 1}}00$$

Related to^51^, CIA and CIW values are below 60% (minor weathering), 60% to 80% (moderate weathering), and 80% to 100% (intensive weathering). In this study, the maximum value of CIA is recorded 79.56% with an average 67.63%, while the maximum value of CIW reached 83.69% with an average 74.67% (Table 1) indicating moderate to intensive chemical weathering of the shale samples of Khatatba Formation (Upper Safa Member).

### Geochemical composition and provenance signals

**Fig. 4:**
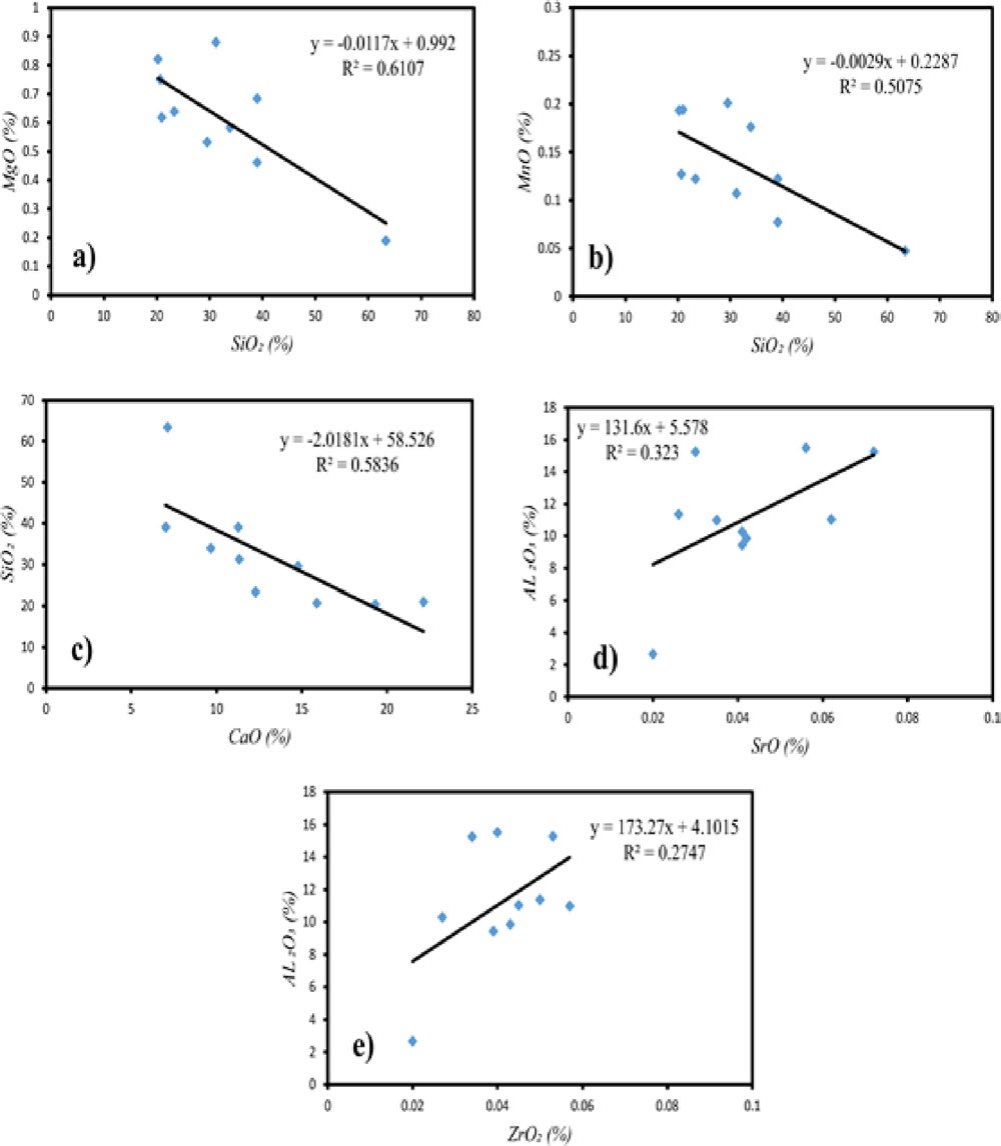
Cross-plots illustrating relationships between major and trace oxides in the Upper Safa Member shale samples: **(a–b)** SiO₂ vs. MgO and MnO, **(c)** CaO vs. SiO_2_ **(d)** SrO vs. Al₂O₃, and **(e)** ZrO_2_ vs. Al_2_O_3_, showing elemental trends linked to provenance and depositional controls.

The major and trace element data provide important insights into sediment origin and depositional processes within the Upper Safa Member. Positive correlations between SiO₂, CaO, MgO, and MnO (Fig. 4) indicate that the sediments were primarily sourced from continental crustal rocks rich in feldspar and clay minerals, supporting a dominantly terrigenous siliciclastic supply to the basin^17,36,50^. Quartz and aluminosilicate contributions reflect strong mechanical and chemical weathering prior to transport into the shallow-marine depositional setting.

The geochemical relationships presented in Fig. 4 should be interpreted with caution, as several of the observed trends exhibit relatively low coefficients of determination (R²), indicating weak correlations between major and trace elements. While general associations between SiO₂, Al₂O₃, and other oxides may suggest a contribution from terrigenous siliciclastic sources, these relationships alone are insufficient to robustly constrain sediment provenance. The scatter in the data likely reflects the combined effects of lithological heterogeneity, diagenetic alteration, and mixed sediment sources. Therefore, the inferred provenance signals are considered indicative rather than definitive, and more reliable constraints would require stronger geochemical correlations or additional proxies such as trace element ratios, rare earth element patterns, or isotopic data.

### Organic matter richness and preservation

**Fig. 5:**
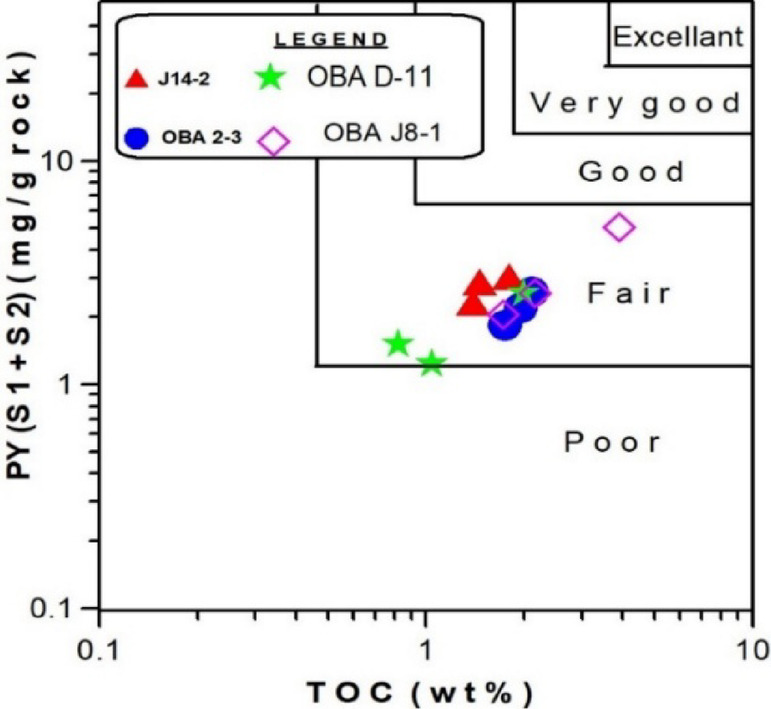
TOC versus Potential Yield (S1 + S2) for Khatatba Formation (Upper Safa Member) shales, illustrating fair to good hydrocarbon generation potential in the analyzed wells. .

The TOC data for the Upper Safa Member exhibit values between 0.50 and 3.90 wt% (Table 2; Fig. 5), demonstrating that these shales contain moderate to excellent quantities of preserved organic matter. Most samples exceed the widely accepted threshold of 0.5 wt% required for effective source rock classification and several intervals surpass 2 wt%, characteristics of highly productive petroleum-generating systems^17,44,55^. These findings highlight the potential of the Upper Safa Member to contribute significantly to hydrocarbon generation within the Obaiyed Field.

**Fig. 6:**
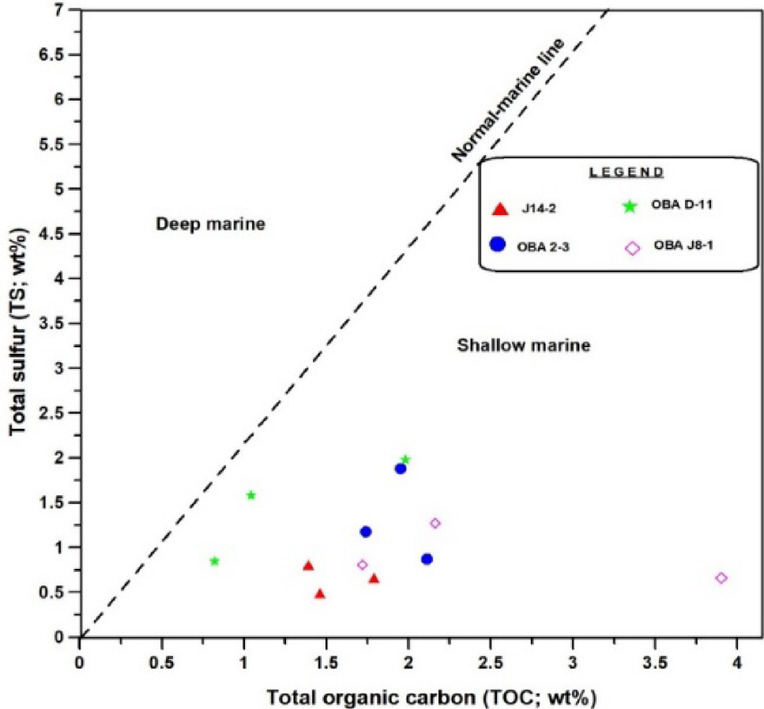
TOC versus TS cross-plot for Khatatba Formation (Upper Safa Member) shale samples, suggesting predominantly shallow-marine depositional conditions in the studied wells.

The positive correlation observed between TOC and TS (Fig. 6) provides additional evidence for favorable depositional conditions, indicating that sulfur incorporation likely occurred under suboxic to dysoxic conditions where reduced bottom-water circulation enhanced organic preservation. Such environments help limit the oxidation of organic material, supporting the retention of hydrogen-rich kerogen within fine-grained sediments^44^. The geochemical data therefore suggest periodic restriction in the depositional setting, which would have promoted the accumulation and preservation of marine and terrestrial organic matter^56,57^. Overall, the richness and preservation of organic matter within the Upper Safa Member align with regional geological interpretations that identify the Khatatba Formation as a key contributor to gas-charged reservoirs throughout the northwestern Western Desert.

The interpretation of redox conditions based on TOC–TS relationships should be considered with caution. Although the positive correlation between TOC and TS suggests enhanced preservation of organic matter under oxygen-restricted conditions, sulfur enrichment may also result from diagenetic processes such as bacterial sulfate reduction or external sulfur inputs, and therefore is not a unique indicator of bottom-water anoxia^44^. In the absence of independent redox-sensitive proxies such as V/(V + Ni), U/Th, or Mo enrichment factors, the depositional environment cannot be confidently classified as anoxic. Accordingly, the studied shale intervals are more appropriately interpreted as having been deposited under **dysoxic to suboxic conditions**, which are sufficient to support organic matter preservation. Future work integrating trace element redox proxies would help to better constrain the paleo-redox conditions of the Khatatba Formation.

### Kerogen type, generation potential, and maturity

**Fig. 7:**
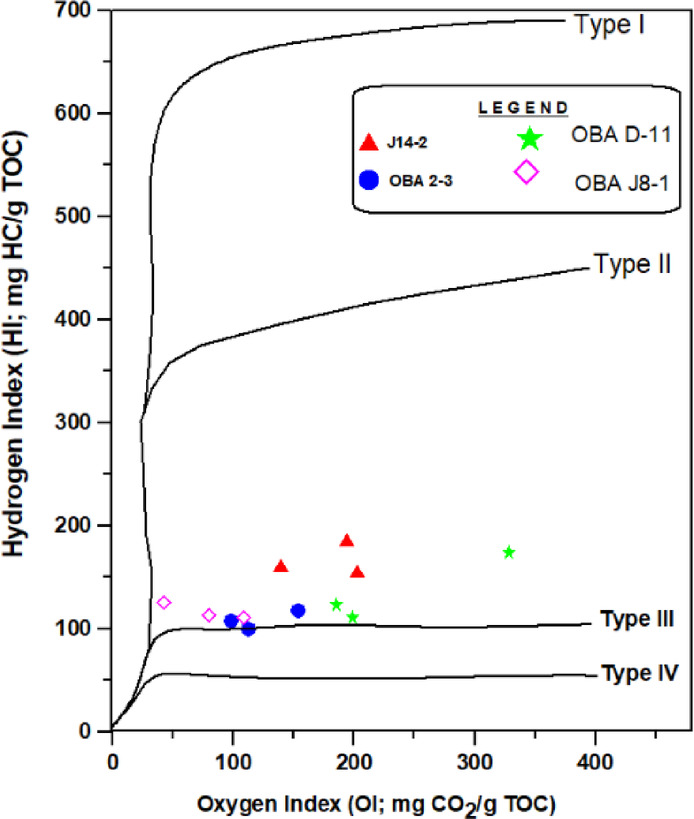
HI–OI diagram for Khatatba Formation (Upper Safa Member) shale samples, showing predominantly mixed Type II/III kerogen with potential for both oil and gas generation.

**Fig. 8:**
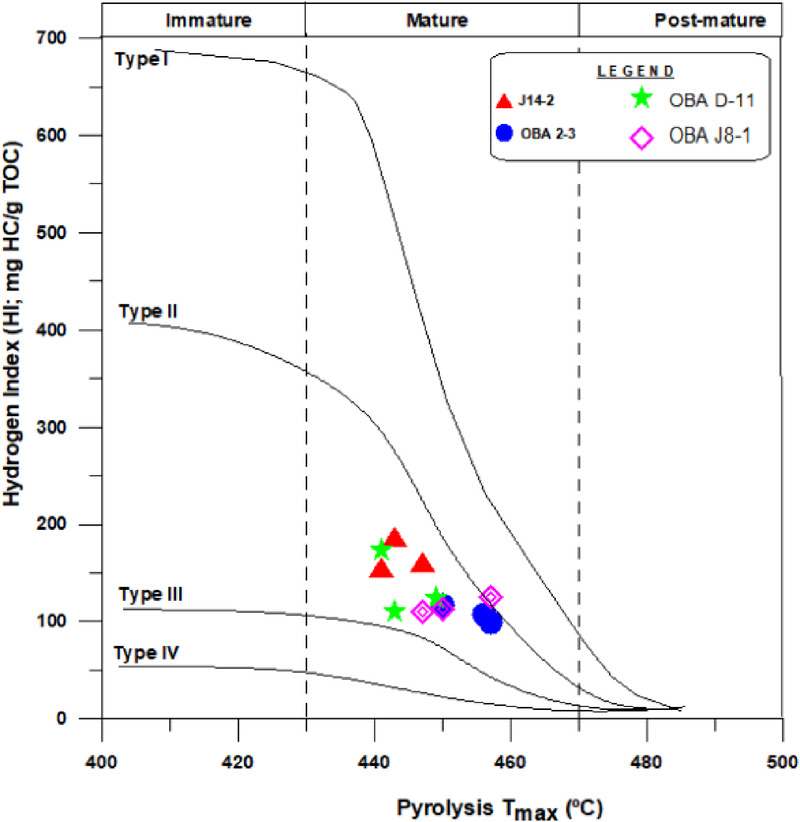
HI versus T_max_ plot for Khatatba Formation (Upper Safa Member) shales, indicating predominantly mature mixed Type II/III kerogen with the capacity for both oil and gas generation.

Rock-Eval pyrolysis data indicate that the Upper Safa Member contains predominantly mixed Type II/III kerogen, as reflected by Hydrogen Index values ranging between 99 and 186 mg HC/g TOC (Fig. 7). This kerogen mixture suggests the combined contribution of marine algal organic matter and terrestrial plant debris, which is capable of generating both oil and gas during thermal evolution^15,18^. The measured Tmax values (441–457 °C; Fig. 8) fall within the established oil generation window, confirming that the organic matter has reached a thermally mature stage conducive to active hydrocarbon generation^3,6,19^.

**Fig. 9:**
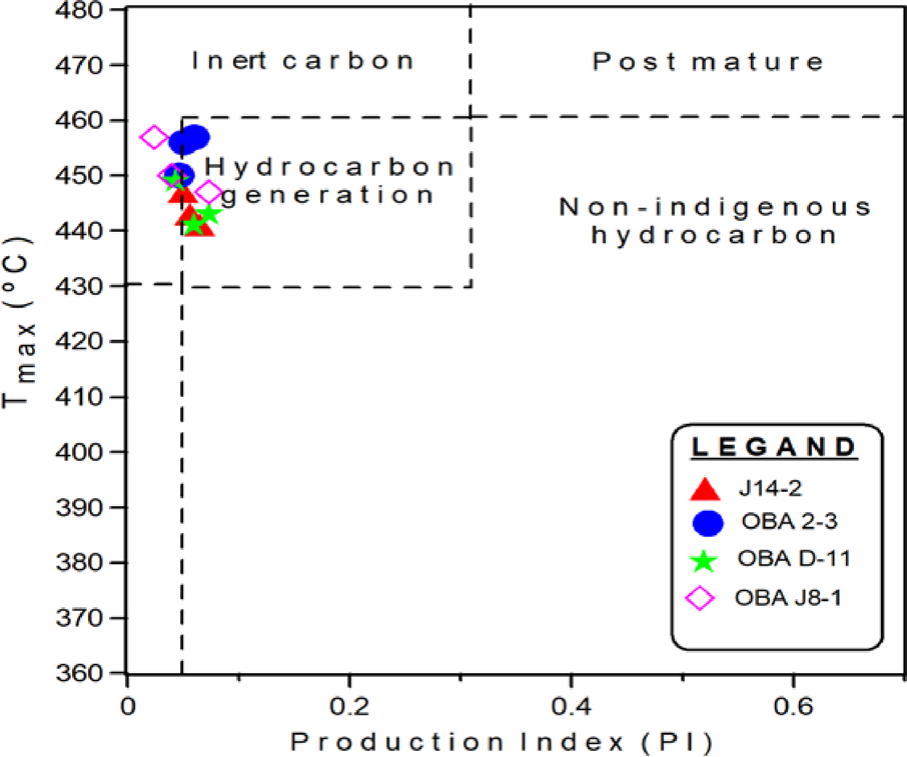
PI versus T_max_ plot for Khatatba Formation (Upper Safa Member) shale samples, indicating thermal maturity within the hydrocarbon generation window. .

Production Index (PI) values remain relatively low (0.02–0.07), which may reflect hydrocarbon loss during sample preparation or early migration from the source interval rather than immaturity (Fig. 9). Potential yield (S1 + S2) values ranging from 1.24 to 5.03 mg HC/g rock indicate fair to good hydrocarbon generative capacity for the studied shales. These results provide strong evidence that the Upper Safa Member has already contributed hydrocarbons to adjacent Safa sandstone reservoirs and continues to act as an important source interval within the Obaiyed petroleum system.

### Insights from log-derived TOC and petroleum system implications

**Fig. 10:**
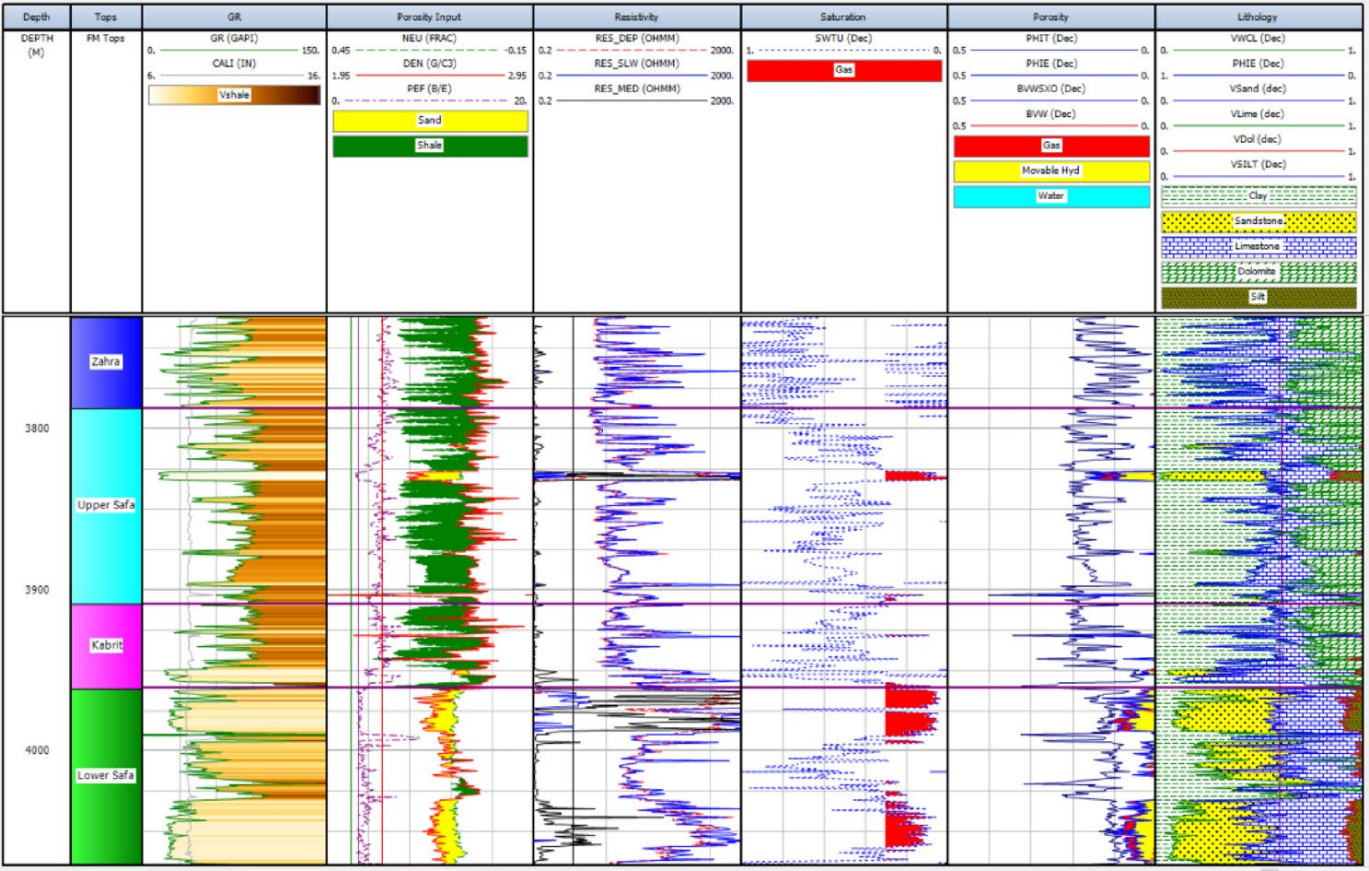
Composite well-log panel at well (OBA D11) showing gamma ray, porosity, resistivity, saturation, and lithology across the Khatatba Formation (Zahra, Upper Safa, Kabrit, and Lower Safa intervals). Clean sandstone units exhibit high resistivity and gas saturation, while shale intervals display elevated GR and characteristic source-rock log responses.

**Fig. 11:**
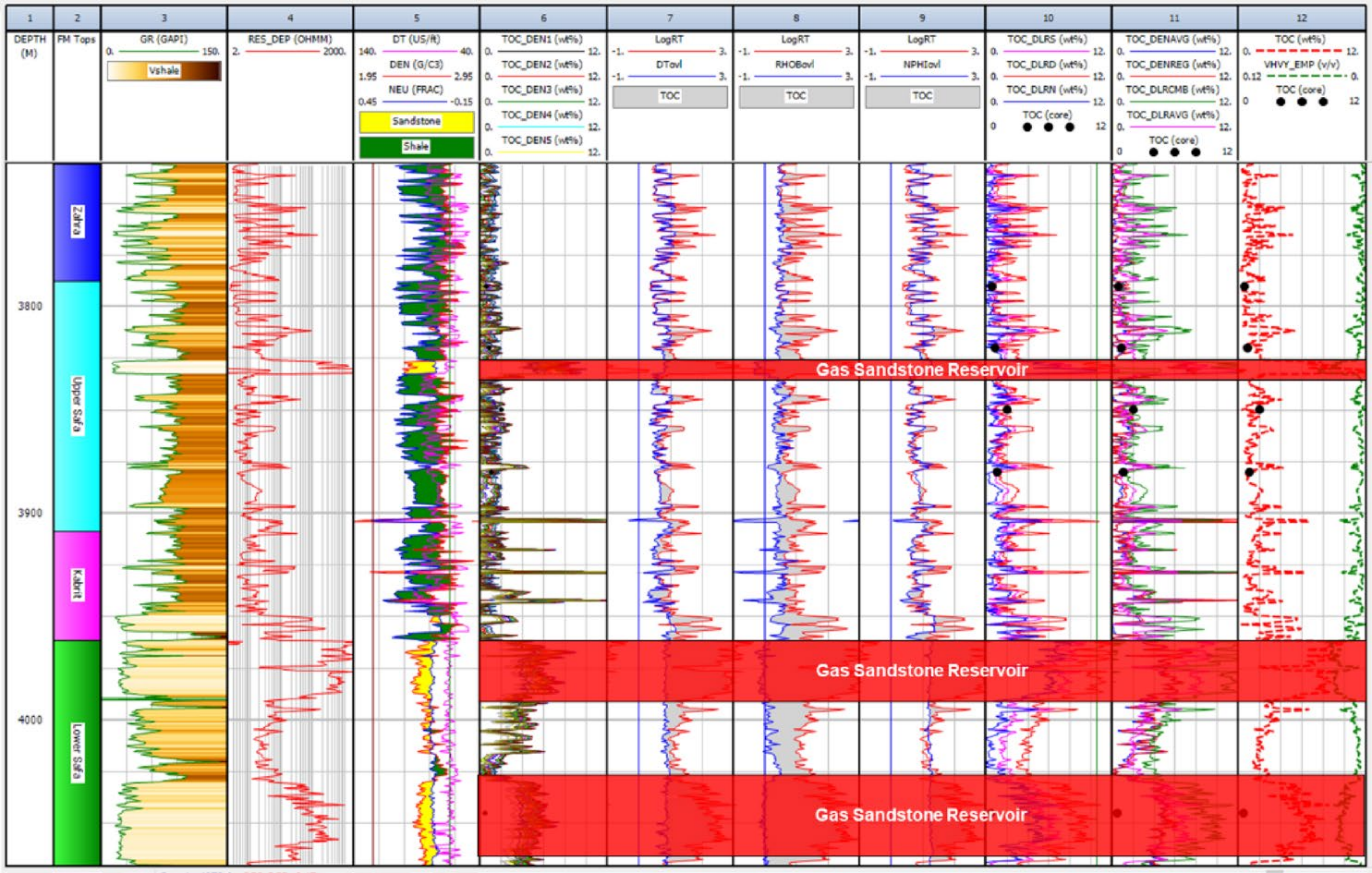
Composite log panel at well (OBA D11) showing multiple log-derived TOC (TOC_DEN, TOC_DLR, TOC_RHOB, TOC_NPHI) calibrated against core TOC for the Khatatba Formation (Zahra, Upper Safa, Kabrit, and Lower Safa intervals). High TOC values correspond to organic-rich shale intervals, whereas sandstone units display low TOC and coincide with gas-bearing reservoir zones.

The wireline-based TOC profiles (Figs. 10 and 11) provide a continuous assessment of organic richness and show strong agreement with laboratory TOC measurements, validating the ΔLogR and multi-parameter TOC transforms used^24,47,48^. High log-derived TOC responses occur within thick shale packages of the Upper Safa Member, confirming its role as the primary source rock interval. Conversely, sandstone-dominated intervals with low TOC correspond to gas-bearing reservoirs, as indicated by high resistivity and neutron-density crossover (Fig. 10). These findings support a self-sourcing petroleum system, where mature Upper Safa shales charge adjacent Upper and Lower Safa sandstone reservoirs vertically within short migration pathways.

Log-derived TOC estimates generated from multiple petrophysical methods demonstrate consistent organic richness trends across the Khatatba Formation, particularly within the Upper Safa Member. The TOC curves derived from density, resistivity, and sonic-based algorithms generally range between 0.5 and 4.0 wt%, aligning well with core-measured values and confirming moderate to very good organic matter enrichment in the shale-prone intervals. Notably, the highest TOC values are observed within the Upper Safa shales between approximately 3820–3900 m depth, where log-based calculations exceed 3 wt% in localized zones, indicating strong hydrocarbon generation potential. In contrast, sandstone-dominated units exhibit lower TOC values (< 1 wt%), reflecting reduced organic content due to higher detrital dilution and enhanced fluid flow. The strong correlation between log-derived and laboratory TOC profiles supports the reliability of the applied methods for continuous TOC prediction in un-cored sections, thereby enhancing source rock characterization in the Obaiyed Field.

Sandstone-dominated intervals—particularly in the Upper and Lower Safa members—exhibit low TOC values and are associated with high resistivity responses, neutron-density crossover, and gas-column signatures, confirming their reservoir nature rather than source potential. The close agreement between core TOC data and log-derived TOC supports the reliability of the petrophysical TOC estimation and provides a continuous high-resolution representation of organic richness through the entire formation. These results highlight that the Upper Safa shales constitute the primary source intervals, while the overlying and underlying sand bodies act as high-quality gas reservoirs.

**Fig. 12:**
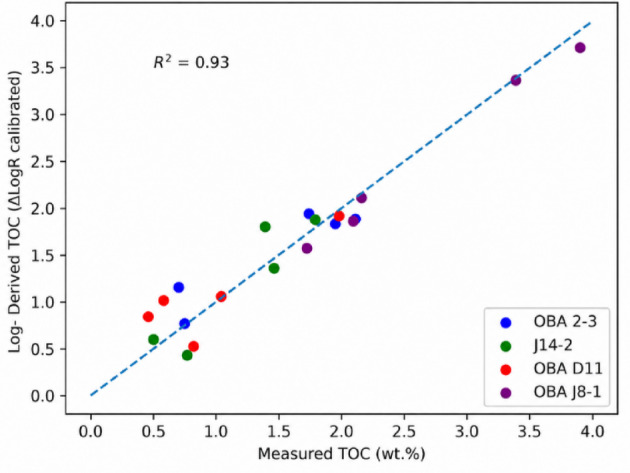
Cross-plot of measured TOC versus Log- Derived (ΔLogR-calibrated) TOC for different wells in the Obaiyed Field. Data points are color-coded by well, showing consistent agreement between measured and log-derived TOC values. The high R² value indicates strong predictive performance of the calibrated model.

The calibration of log-derived TOC against laboratory measurements demonstrates a high level of accuracy and reliability for the applied workflow. The cross-plot between measured TOC and predicted (ΔLogR-calibrated) TOC yields a strong linear relationship with a coefficient of determination (R² = 0.93), indicating excellent predictive performance across the studied wells (Fig. 12). The majority of data points cluster closely around the 1:1 line, confirming that the log-derived estimates effectively reproduce the magnitude and vertical variability of organic richness. This agreement is consistent across all wells, suggesting that the calibration is robust despite variations in lithology and reservoir conditions. Slight deviations at lower TOC values are observed but remain within acceptable error margins and are attributed to lithological effects and uncertainties associated with ditch-cutting samples. Overall, these results validate the use of the ΔLogR method, when properly calibrated, as a reliable tool for continuous TOC estimation in the Khatatba Formation.

**Fig. 13:**
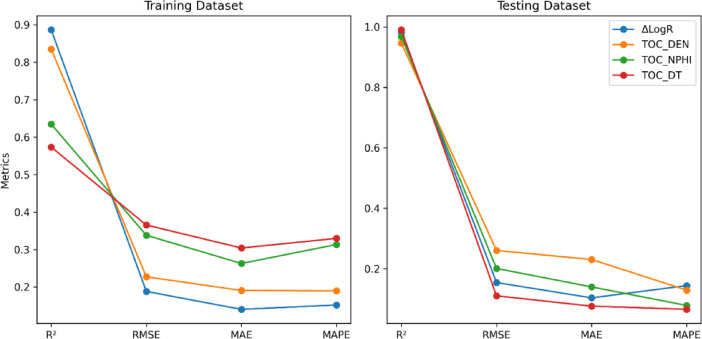
Comparison of predictive performance for different TOC estimation methods using training and testing datasets. The ΔLogR-based model shows consistently higher R² values and lower error metrics (RMSE, MAE, and MAPE), indicating superior reliability compared to density-, neutron-, and sonic-based approaches.

The comparative evaluation of TOC prediction methods demonstrates that the ΔLogR-based approach consistently outperforms other petrophysical models across both training and testing datasets (Fig. 13). The ΔLogR method yields the highest coefficient of determination (R²) and the lowest error values (RMSE, MAE, and MAPE), indicating strong agreement with laboratory-measured TOC. In contrast, TOC estimates derived from density, neutron, and sonic logs exhibit comparatively lower R² values and higher prediction errors, reflecting reduced accuracy and greater uncertainty. The consistency of ΔLogR performance across both datasets highlights its robustness and reliability for continuous TOC estimation. These results confirm that, when properly calibrated, the ΔLogR method provides a more accurate representation of organic richness compared to other single-log approaches.

This integrated evaluation aligns with regional studies demonstrating that the Khatatba Formation provides a major contribution to gas accumulations in the Western Desert^10,11,13^. The combination of good organic richness, mature mixed kerogen, and spatial proximity to high-quality reservoirs emphasizes the strategic importance of this unit in Obaiyed Field hydrocarbon generation and entrapment.

### Depositional environment (uncertainty and limitations)

The interpretations of depositional environment, provenance, and preservation conditions of the Khatatba Formation (Upper Safa Member) are based on integrated mineralogical, geochemical, and organic geochemical datasets, and should therefore be regarded as consistent with the available data but not uniquely constrained. The dominance of kaolinite, together with quartz and minor calcite, supports deposition in a siliciclastic system influenced by continental input with intermittent marine conditions, which is compatible with a shallow-marine setting^18,21,41^. However, kaolinite-rich assemblages may also form under different climatic and diagenetic conditions, and thus cannot alone provide definitive evidence for specific paleoclimatic or depositional scenarios^41^. Similarly, the major and trace element relationships (e.g., SiO₂–Al₂O₃ trends and CaO–SrO associations) suggest mixed siliciclastic and minor carbonate contributions, but the relatively weak correlations limit robust interpretation of provenance without additional constraints such as rare earth elements or isotopic data^17,36,50,58,59^.

The preservation conditions inferred from TOC–TS relationships indicate deposition under oxygen-restricted conditions favorable for organic matter accumulation; however, sulfur enrichment may also reflect diagenetic processes such as bacterial sulfate reduction and is not a definitive indicator of bottom-water anoxia^44^. In the absence of independent redox-sensitive proxies (e.g., V/(V + Ni), U/Th), the depositional environment is more appropriately interpreted as dysoxic to suboxic rather than strictly anoxic. Furthermore, the variability in TOC (0.50–3.90 wt%) and Rock-Eval parameters reflects heterogeneity in organic matter input and preservation efficiency, which is typical of mixed marine–terrigenous systems^17,18^. Accordingly, the interpretations presented in this study are expressed cautiously and are intended to represent the most plausible geological scenario consistent with the available data, while acknowledging that additional geochemical proxies would further refine depositional and paleoenvironmental reconstructions.

## Conclusions

This study presents an integrated mineralogical, geochemical, and petrophysical evaluation of the Middle Jurassic Khatatba Formation in the Obaiyed Field, Western Desert, Egypt, with particular focus on the Upper Safa Member as the principal source rock interval. Mineralogical analyses indicate a kaolinite-rich siliciclastic assemblage with quartz and minor calcite, suggesting mixed continental and marine influence, although these signatures are interpreted cautiously because they may also reflect diagenetic processes and source rock variations. The maximum CIA and CIW values reached 79.56% and 83.69%, respectively, indicating moderate to intensive chemical weathering of the Upper Safa Member shale samples. Geochemical results show that SiO₂, Al₂O₃, and CaO are the dominant oxides, reflecting a mixed siliciclastic–carbonate system with predominantly terrigenous input. Organic geochemical analyses indicate moderate to very good organic richness, with TOC values ranging from 0.50 to 3.90 wt% (average ~ 1.8 wt%). Rock-Eval pyrolysis reveals predominantly mixed Type II/III kerogen with HI values between 99 and 186 mg HC/g TOC, while Tmax values of 441–457 °C confirm thermal maturity within the hydrocarbon generation window and indicate gas-prone source rock characteristics.

The integration of calibrated log-derived TOC with laboratory measurements demonstrates strong agreement (R² = 0.93), confirming the reliability of the applied workflow for continuous TOC characterization and highlighting vertical heterogeneity in organic richness within the Upper Safa Member. The TOC–TS relationship suggests oxygen-restricted depositional conditions favorable for organic matter preservation; however, in the absence of independent redox-sensitive proxies, the depositional environment is more appropriately interpreted as dysoxic to suboxic rather than fully anoxic. The close stratigraphic association between mature organic-rich shales and adjacent gas-bearing sandstone reservoirs supports a self-sourcing petroleum system characterized by short-distance vertical migration. Overall, the Upper Safa Member is interpreted as a mature, gas-prone source rock with fair to good hydrocarbon generation potential. The integrated workflow and datasets presented in this study provide a reproducible framework for continuous source rock characterization and a valuable baseline reference for future studies of the Khatatba Formation as an unconventional shale gas reservoir, particularly for rock mechanics, geomechanics, and hydraulic fracturing applications.

## Data Availability

The datasets used and/or analysed during the current study available from the corresponding author on reasonable request.

## References

[1] Makky, A. F. et al. Source rock evaluation of some upper and lower Cretaceous sequences, West Beni Suef Concession, Western Desert, Egypt. *Egypt. J. Petroleum*. **23** (1), 135–149. 2014/03/01/ (2014).

[2] Sarhan, M. A. Seismic – wireline logs sequence stratigraphic analyses and geologic evolution for the Upper Cretaceous succession of Abu Gharadig basin, Egypt. *Journal of African Earth Sciences* **129**, 469–480. 10.1016/j.jafrearsci.2017.02.004 (2017).

[3] Sarhan, M. A. Geophysical appraisal for the sandy levels within Abu Roash C and E members in Abu Gharadig Field, Western Desert, Egypt. *Journal of Petroleum Exploration and Production Technology* **11**(3), 1101–1122. 10.1007/s13202-021-01107-3 (2021).

[4] Sarhan, M. A. Geophysical assessment and hydrocarbon potential of the Cenomanian Bahariya reservoir in the Abu Gharadig Field, Western Desert, Egypt. *Journal of Petroleum Exploration and Production Technology* **11**(11), 3963–3993. 10.1007/s13202-021-01289-w (2021).

[5] Abdel-Fattah, M., Hanafy, M., Hamdan, H. & Attia, T. Integrated 3D Reservoir Modeling and Tectonic Evaluation of the Upper Cretaceous Bahariya Formation in the Burg El Arab Area, Egypt: Implications for Hydrocarbon Exploration and Production Strategies. *Egypt. J. Petroleum*. **33** (2), 8 (2024).

[6] Abdel-Fattah, M. I. et al. Oil-source correlation and Paleozoic source rock analysis in the Siwa Basin, Western Desert: insights from well-logs, Rock-Eval pyrolysis, and biomarker data. *Energy Geoscience*. **5** (3), 100298 (2024).

[7] Shehata, A. A. Cenomanian–Turonian depositional history of a post–Gondwana rift succession in the West Beni Suef Basin, Egypt. *Journal of African Earth Sciences* **150**, 783–798. 10.1016/j.jafrearsci.2018.10.006 (2019).

[8] Sakran, S., Shehata, A. A., Osman, O. & El-Sherbiny, M. Superposed tectonic regimes in west Beni Suef basin, Nile Valley, Egypt: Implications to source rock maturation and hydrocarbon entrapment. *Journal of African Earth Sciences* **154**, 1–19. 10.1016/j.jafrearsci.2019.03.010 (2019).

[9] Abdel-Fattah, M. I., Attia, T. E., Abd El-Aal, M. H. & Hanafy, M. I. Seismic interpretation of the Late Albian-Early Cenomanian Bahariya reservoirs of Burg El Arab oil field for tectonic evaluation: a case study from Western Desert, Egypt. *Arab. J. Geosci.* **14** (5), 412 (2021).

[10] Abdel-Fattah, M. I. Impact of depositional environment on petrophysical reservoir characteristics in Obaiyed Field, Western Desert, Egypt. *Arab. J. Geosci.* **8** (11), 9301–9314 (2015).

[11] Shehata, A. A., Abdel-Fattah, M. I., Hamdan, H. A. & Sarhan, M. A. Seismic interpretation and sequence stratigraphic analysis of the Bahariya Formation in the South Umbaraka oilfields (Western Desert, Egypt): Insights into reservoir distribution, architecture, and evaluation. *Geomechanics and Geophysics for Geo-Energy and Geo-Resources* **9**(1), 135. 10.1007/s40948-023-00673-6 (2023).

[12] Shehata, A. A., Sarhan, M. A., Abdel-Fattah, M. I. & Mansour, S. Geophysical assessment for the oil potentiality of the Abu Roash “G” reservoir in West Beni Suef Basin, Western Desert, Egypt. *Journal of African Earth Sciences* **199**, 104845. 10.1016/j.jafrearsci.2023.104845 (2023).

[13] Reda, M., Abdel-Fattah, M. I., Fathy, M. & Bakr, A. Integrated analysis of source rock evaluation and basin modelling in the Abu Gharadig Basin, Western Desert, Egypt: Insights from pyrolysis data, burial history, and trap characteristics. *Geol. J.* **59** (4), 1416–1443. 10.1002/gj.4938 (2024).

[14] Soliman, S. R., Salama, Y. F., El-Sayed, M. I., Abdel-Fattah, M. I. & Abd-Allah, Z. M. Assessment of mineralogical and geochemical composition of Oligocene/Eocene black shale deposits in Beni Suef Area, Egypt. *Advances in Materials Science and Engineering* **2022**, 1. 10.1155/2022/1606431 (2022).

[15] Gharib, A. F. et al. Organic matter characteristics and hydrocarbon generation potential of the Middle Jurassic–Lower Cretaceous succession in the Mesopotamian Foredeep Basin, Iraq. *International Journal of Earth Sciences* **113**(8), 2163–2187. 10.1007/s00531-024-02434-6 (2024).

[16] Abdel-Fattah, M. I. et al. *Integrated Approach and Insights into Shale Gas Potential: Geological, Petrophysical, Geochemical, and Geomechanical Evaluation of the Khatatba Formation* (Western Desert, Egypt), Petroleum Research, 2025).

[17] Peters, K. E. Guidelines for Evaluating Petroleum Source Rock Using Programmed Pyrolysis1, *AAPG Bulletin,* **70**(3), 318–329, (1986). 10.1306/94885688-1704-11d7-8645000102c1865d

[18] El Diasty, W. S. Khatatba Formation as an active source rock for hydrocarbons in the northeast Abu Gharadig Basin, north Western Desert, Egypt. *Arab. J. Geosci.*, **8**, 4, pp. 1903–1920, 2015/04/01 2015, 10.1007/s12517-014-1334-x

[19] Mahdi, A., Al-Beyati, F., Tarif, M., Shendi, E. A. & Abdel-Fattah, M. Palynofacies and Paleoenvironment investigation of the Hauterivian–Early Aptian Ratawi and Zubair formations, Balad oilfield, Central Iraq. *Tikrit J. Pure Sci.* **24**, 74–80. 10.25130/tjps.24.2019.111 (01/01 2019).

[20] Mahdi, A. Q., Abdel-Fattah, M. I., Radwan, A. E. & Hamdan, H. A. An integrated geochemical analysis, basin modeling, and palynofacies analysis for characterizing mixed organic-rich carbonate and shale rocks in Mesopotamian Basin, Iraq: Insights for multisource rocks evaluation. *J. Petrol. Sci. Eng.* **216**, 110832. 2022/09/01/ (2022).

[21] Shalaby, M. R., Hakimi, M. H. & Abdullah, W. H. Organic geochemical characteristics and interpreted depositional environment of the Khatatba Formation, northern Western Desert. *Egypt. AAPG Bull.* **96** (11), 2019–2036. 10.1306/04181211178 (2012).

[22] Abdel-Fattah, M. I., Metwalli, F. I. & El Sayed, I. M. Static reservoir modeling of the Bahariya reservoirs for the oilfields development in South Umbarka area, Western Desert, Egypt. *J. Afr. Earth Sc.* **138**, 1–13 (2018).

[23] Sarhan, M. A. Seismic delineation and well logging evaluation for albian Kharita Formation, South West Qarun (SWQ) field, Gindi Basin, Egypt. *J. Afr. Earth Sc.*, **158**, p. 103544, 2019/10/01/ 2019, doi: 10.1016/j.jafrearsci.2019.103544

[24] Passey, Q. R., Creaney, S., Kulla, J. B., Moretti, F. J. & Stroud, J. D. A Practical Model for Organic Richness from Porosity and Resistivity Logs1, *AAPG Bulletin,* **74**(12), 1777–1794, (1990). 10.1306/0c9b25c9-1710-11d7-8645000102c1865d

[25] Kamali, M. R. & Allah Mirshady, ". Total organic carbon content determined from well logs using ΔLogR and neuro fuzzy techniques. *Journal of Petroleum Science and Engineering* **45**(3), 141–148. 10.1016/j.petrol.2004.08.005 (2004).

[26] De, S., Varma, A. K. & Sengupta, D. Recent Advances in Well Logging Techniques for Exploration of Shale Reservoirs, in Unconventional Shale Gas Exploration and Exploitation: Current Trends in Shale Gas Exploitation, A. Boruah, S. Verma, and S. S. Ganguli Eds. Cham: Springer International Publishing, pp. 49–67. (2024).

[27] Aliakbardoust, E., Adabi, M. H., Kadkhodaie, A., Harris, N. B. & Chehrazi, A. Integration of well logs and seismic attributes for prediction of thermal maturity and TOC content in the Kazhdumi Formation (central Persian Gulf basin), JAG, **222**, 105319, (2024).

[28] Proestakis, E. & Fabricius, I. L. Log Interpretation for Petrophysical and Elastic Properties of Fine-Grained Sedimentary Rocks. *Petrophysics - SPWLA J.* **66**, 705–727. 10.30632/PJV66N5-2025a1 (2025).

[29] Shalaby, M. R., Hakimi, M. H. & Abdullah, W. H. Diagenesis in the Middle Jurassic Khatatba Formation sandstones in the Shoushan Basin, northern Western Desert, Egypt. *Geological Journal* **49**(3), 239–255. 10.1002/gj.2512 (2014).

[30] Rossi, C., Marfil, R., Ramseyer, K. & Permanyer, A. Facies-related diagenesis and multiphase siderite cementation and dissolution in the reservoir sandstones of the Khatatba Formation, Egypt’s Western Desert. *Journal of Sedimentary Research* **71**(3), 459–472. 10.1306/2dc40955-0e47-11d7-8643000102c1865d (2001).

[31] Mousa, D. A., Abuhagaza, A. A., Mahdi, A. Q., Gentzis, T. & Makled, W. A. Assessment of the hydrocarbon potential in the black shales of the Jurassic Khatatba Formation and generated hydrocarbons, North Western Desert, Egypt: Depositional mechanism of organic rich rocks related to syn-rift differential subsidence, *Marine and Petroleum Geology*, **167**, 106975, 2024/09/01/ (2024). 10.1016/j.marpetgeo.2024.106975

[32] Mostafa, A., El-Barkooky, A. & Hammed, M. Structural geometry and tectonic evolution of an Early Cretaceous rift crossing the Nile Valley in Upper Egypt. *Mar. Pet. Geol.* **153**, 106289. 10.1016/j.marpetgeo.2023.106289 (2023).

[33] Yousef, M., Hamimi, Z., Heneish, A., Hagag, W. & Anan, T. Phanerozoic Structural Setting and Tectonic Evolution of Egypt. In *The Phanerozoic Geology and Natural Resources of Egypt* (ed. Hamimi, Z.) 27–82 (Springer International Publishing, 2023).

[34] Shaw, D. B. & Weaver, C. E. The mineralogical composition of shales. *J. Sediment. Res.* **35** (1), 213–222. 10.1306/74d71221-2b21-11d7-8648000102c1865d (1965).

[35] Abanda, P. A. & Hannigan, R. E. Effect of diagenesis on trace element partitioning in shales, *Chemical Geology*, **230**(1–2), 42–59, (2006).

[36] Scholz, F. Identifying oxygen minimum zone-type biogeochemical cycling in Earth history using inorganic geochemical proxies. *Earth Sci. Rev.*, **184**, 08/01 2018, 10.1016/j.earscirev.2018.08.002

[37] Worden, R. H. et al. Chlorite in sandstones, *Earth-Science Reviews*, **204**. 103105, (2020). 10.1016/j.earscirev.2020.103105

[38] Qiu, Z., Liu, B., Lu, B., Shi, Z. & Li, Z. Mineralogical and petrographic characteristics of the Ordovician-Silurian Wufeng-Longmaxi Shale in the Sichuan Basin and implications for depositional conditions and diagenesis of black shales. *Mar. Pet. Geol.*, **135**, p. 105428, 2022/01/01/ 2022, doi: 10.1016/j.marpetgeo.2021.105428

[39] Yang, H. et al. Characteristics of Mineralogy, Lithofacies of Fine-Grained Sediments and Their Relationship with Sedimentary Environment: Example from the Upper Permian Longtan Formation in the Sichuan Basin, *Energies*, **14**(12), 3662 10.3390/en14123662

[40] Khan, D. et al. Mineralogical and geochemical characterization of lacustrine calcareous shale in Dongying Depression, Bohai Bay Basin: Implications for paleosalinity, paleoclimate, and paleoredox conditions, *Geoch*, **83**(3), 125978, (2023). 10.1016/j.chemer.2023.125978

[41] Thiry, M. Palaeoclimatic interpretation of clay minerals in marine deposits: An outlook from the continental origin. *Earth-Science Reviews* **49**, 201–221 (2000).

[42] Bernard, B. B., Bernard, H. & Brooks, J. M. *Determination of carbon forms in sediments* (Texas A&M University, 1995).

[43] Rosenberg, Y. O. et al. Study of thermal maturation processes of sulfur-rich source rock using compound specific sulfur isotope analysis, *Organic Geochemistry,* **112**, 59–74, (2017). 10.1016/j.orggeochem.2017.06.005

[44] Abd El Gawad, E. A. et al. Relationship between cyclic patterns of organic matter concentrations and their paleoenvironmental controls in the Upper Cretaceous rocks in Abu El Gharadig basin, Egypt: Cyclo-sequence stratigraphic framework. *Mar. Pet. Geol.* **136**, 105474 (2022).

[45] Abd-Allah, Z. M., Abdullah, W. H. & Abdel-Fattah, M. I. Assessment of Eocene, Paleocene and Cretaceous source rocks in the West Feiran area, offshore Gulf of Suez, Egypt. *J. Petrol. Sci. Eng.* **180**, 756–772 (2019).

[46] Reda, M. et al. Integrated petrophysical analysis, seismic interpretation, and 3D static reservoir modelling for rock typing and geomechanical characterisation of Cretaceous reservoirs in the Arcadia and Dorra oil fields, Western Desert, Egypt. *Geological Journal*10.1002/gj.70122 (2025).

[47] Lai, J. et al. Well-log prediction of TOC: A comprehensive review. *Earth Sci. Rev.* **258**, 104913 (2024).

[48] Nyakilla, E. E. et al. Evaluation of source rock potentiality and prediction of total organic carbon using well log data and integrated methods of multivariate analysis, machine learning, and geochemical analysis. *Natural Resources Research* **31**(1), 619–641. 10.1007/s11053-021-09988-1 (2022).

[49] Wang, Y. et al. Geological Characteristics of Shale Gas in Different Strata of Marine Facies in South China. *J. Earth Sci.* **32**(4), 725–741. 10.1007/s12583-020-1104-5 (2021) (**2021/08/01**).

[50] Ray, E. & Paul, D. Major and Trace Element Characteristics of the Average Indian Post-Archean Shale: Implications for Provenance, Weathering, and Depositional Environment. *ACS Earth Space Chem.* **5**(5), 1114–1129. 10.1021/acsearthspacechem.1c00030 (2021) (**2021/05/20**).

[51] Fedo, C. M., Nesbitt, H. W. & Young, G. M. Unraveling the effects of potassium metasomatism in sedimentary rocks and paleosols. *Geology* **23**, 921–924 (1995).

[52] Nesbitt, H. W. & Young, G. M. Formation and diagenesis of weathering profiles. *J. Geol.* **97**, 129–147 (1989).

[53] Harnois, L. The CIW index: A new chemical index of weathering. *Sed. Geol.* **55**, 319–322 (1988).

[54] McLennan, S. M., Hemming, S., McDaniel, D. K. & Hanson, G. N. *Geochemical approaches to sedimentation, provenance, and tectonics* (Geological Society of America Special Paper, 1993).

[55] Woltz, C. R. et al. Total organic carbon and the preservation of organic-walled microfossils in Precambrian shale. *Geology* **49**(5), 556–560. 10.1130/g48116.1 (2020).

[56] Chen, K. et al. Depositional-diagenetic process and their implications for pore development of Wufeng-Longmaxi shales in the Jiangdong block, Fuling shale gas field, SW China. *Marine and Petroleum Geology* **151**, 106177. 10.1016/j.marpetgeo.2023.106177 (2023).

[57] Zhao, W. et al. Role of preservation conditions on enrichment and fluidity maintenance of medium to high maturity lacustrine shale oil. *Pet. Explor. Dev.* **52**(1), 1–16. 10.1016/S1876-3804(25)60001-2 (2025).

[58] Soliman, S. R. & Salama, Y. F. Integration of subsurface and outcrop data to evaluate the mineralogy, geochemistry, and hydrocarbon potentiality of Duwi black shale formation at Kharga and Dakhla depressions, Western Desert, Egypt. *Scientific African*10.1016/j.sciaf.2026.e03239 (2026).

[59] Soliman, S. R. et al. Paleoclimate and Depositional Controls on Organic Carbon Storage and Sustainable Unconventional Resource Potential of Late Cretaceous–Paleocene Black Shales, Dakhla Basin, Egypt*. Sustain.* **18** (5), 2332. 10.3390/su18052332 (2026).

